# Change Over Time in Linguistic Acculturation: A Comparison Between Cuban and Non-Cuban Hispanic Immigrants

**DOI:** 10.1093/geroni/igaa057.1073

**Published:** 2020-12-16

**Authors:** David Chiriboga, Nan Sook Park, Yuri Jang, Victor Molinari

**Affiliations:** 1 University of South Florida, Tampa, Florida, United States; 2 University of Southern California, Los Angeles, California, United States

## Abstract

While acculturation and its implications for mental health have received extensive attention in studies with older immigrant populations, change over time in acculturation less so. This paper examines change over a two-year period in levels of linguistic acculturation among Cuban (n = 186) and non-Cuban Hispanic (n = 116) immigrants living in Florida. Data came from the waves of the Survey of Older Floridian (SOF), conducted using a statewide sampling frame based on population densities, with a computer-assisted telephone interview strategy. Calls were initiated through random digit dialing, and a follow-up interview was added approximately two years later. Measures included six acculturation items, one dealing with the participant’s language preference for the interview itself and five items dealing with language ability and usage. Results indicated that non-Cuban Hispanics were significantly more acculturated than Cuban Hispanics, and that at least 75% of Wave 2 acculturation was accounted for by Wave 1 status. After controlling for initial levels of acculturation, those who at first interview reported more economic problems and those reporting fewer depressive symptoms were more acculturated at follow-up. It was concluded that while linguistic acculturation is relatively stable, interventions to increase acculturation have implications for mental health service utilization.

